# Broccoli Microgreens: A Mineral-Rich Crop That Can Diversify Food Systems

**DOI:** 10.3389/fnut.2017.00007

**Published:** 2017-03-23

**Authors:** Carolyn F. Weber

**Affiliations:** ^1^Department of Biological Sciences, Idaho State University, Pocatello, ID, USA

**Keywords:** microgreens, food systems, minerals, urban agriculture, distributed agriculture, sustainability

## Abstract

Current malnourishment statistics are high and are exacerbated by contemporary agricultural practices that damage the very environments on which the production of nutritious food depends. As the World’s population grows at an unprecedented rate, food systems must be revised to provide adequate nutrition while minimizing environmental impacts. One specific nutritional problem that needs attention is mineral (e.g., Fe and Zn) malnutrition, which impacts over two-thirds of the World’s people living in countries of every economic status. Microgreens, the edible cotyledons of many vegetables, herbs, and flowers, is a newly emerging crop that may be a dense source of nutrition and has the potential to be produced in just about any locale. This study examined the mineral concentration of broccoli microgreens produced using compost-based and hydroponic growing methods that are easily implemented in one’s own home. The nutritional value of the resulting microgreens was quantitatively compared to published nutritional data for the mature vegetable. Nutritional data were also considered in the context of the resource demands (i.e., water, fertilizer, and energy) of producing microgreens in order to gain insights into the potential for local microgreen production to diversify food systems, particularly for urban areas, while minimizing the overall environmental impacts of broccoli farming. Regardless of how they were grown, microgreens had larger quantities of Mg, Mn, Cu, and Zn than the vegetable. However, compost-grown (C) microgreens had higher P, K, Mg, Mn, Zn, Fe, Ca, Na, and Cu concentrations than the vegetable. For eight nutritionally important minerals (P, K, Ca, Mg, Mn, Fe, Zn, and Na), the average C microgreen:vegetable nutrient ratio was 1.73. Extrapolation from experimental data presented here indicates that broccoli microgreens would require 158–236 times less water than it does to grow a nutritionally equivalent amount of mature vegetable in the fields of California’s Central Valley in 93–95% less time and without the need for fertilizer, pesticides, or energy-demanding transport from farm to table. The results of this study suggest that broccoli microgreens have the potential to be a rich source of minerals that can be produced by individuals, even in urban settings, providing better access to adequate nutrition.

## Introduction

The strong dependence of human nutrition on the environmental sustainability of crop production has come into focus as problem-solving efforts work to identify mechanisms to feed the World’s rapidly growing population (1). Current malnourishment statistics are high and contemporary agricultural practices are a dominant force in damaging the very environments on which the production of nutritious food depends (1, 2). In the U.S., food production utilizes 50% of land and is responsible for 80% of total freshwater consumption (3), which occurs at a rate that is faster than aquifer recharge in some regions. Food production also depends heavily of fertilizer and pesticide application, which is adversely impacting ecosystem biodiversity (2). Additionally, cultivation is increasingly focused on the mass production of fewer staple crops. This reduces the nutritional value of the average diet and makes food production less resilient to environmental change (4, 5), should it be the demise of one or more of these relatively few crops. Therefore, simply upscaling current agricultural practices to increase crop yields is not a viable solution for feeding the World’s population. It is a priority to establish dietary guidelines that satisfy human nutritional requirements with a diversity of foods that can be produced with minimized environmental impact (6–8); this is key to ensuring socioeconomic and sociocultural prosperity into the future (2).

Achieving such developments requires revising food systems. Food systems are comprised of not only activities associated with food production but also those associated with food processing, transport, consumption, and governance of the above named. In addition to the flaws in food production methods discussed above, 40% of the food produced is never consumed, comprises the largest component of municipal waste, and is responsible for a large fraction of annual methane emissions in the U.S. (3). Much of this food is transported over long distances from farms to urban centers, which consumes 10% of the total energy budget in the U.S. (3) and contributes to food waste as it spoils or is contaminated enroute (2). Reliance on these long food chains threatens food security in urban areas, where over 54% of the World’s population is concentrated (9), as it puts sustenance for their populations at the mercy of natural and anthropogenic disasters in distant locations. The collective makes current food systems vulnerable to the environmental changes they also contribute to.

With respect to nutrition, the flaws in food systems create a dichotomous problem of excess and insufficiency. This is exemplified by one-third of the world’s people being overweight and/or undernourished (2, 8, 10). This problem impacts countries of every economic status (10). The reliance of urban populations on long food chains limits accessibility to produce that has short shelf lives and, therefore, poor transportability, and increases dependence on heavily processed and packaged foods; this creates “food deserts” in urban areas in which people do not have ready access to a complete compliment of required nutrients (11). However, even the fresh produce that does reach its destination has likely lost substantial nutritional value during transport (12).

One specific nutritional problem that is common in developed and developing countries is mineral malnutrition. Over 60, 30, and 15% of the World’s seven billion people are Fe-, Zn-, and Se-deficient, respectively (13). Rates of mineral malnutrition are especially high in Asia and Africa (14), where soil degradation is especially severe and has significantly decreased the nutritional value of crops (15). However, mineral malnutrition is considered to be one of the most important global challenges to mankind that can be prevented (16) and is one of the Millennium Development Goals (14). Current efforts to mitigate mineral malnourishment are focused on developing biofortification methods (13) and genetically engineering crops for maximal nutrient uptake (17).

However, a newly emerging crop that may be a dense source of nutrition in the absence of biofortification and genetic engineering and has the potential to be produced in just about any locale is microgreens. Microgreens are edible seedlings that are usually harvested 7–14 days after germination when they have two fully developed cotyledon leaves (18). A wide variety of herbs (e.g., basil, cilantro), vegetables (e.g., radish, broccoli, and mesclun) and even flowers (e.g., sunflowers) are grown as microgreens. Microgreens are generally more flavorful, some of them quite spicy, than their mature counterparts and have grown in popularity among culinary artists for adding texture and flavor accents to salads, sandwiches, and other dishes (19, 20). The increasing culinary demand as well as the ease with which microgreens can be grown, even by inexperienced gardeners in urban settings, has piqued interests in growing and eating them. Interest in microgreens has also been generated by popular websites (21) touting the findings of Xiao et al. (18), which indicate that microgreens may have 4–40 times the amount of some nutrients and vitamins as the vegetables a mature plant would produce. However, Xiao et al. (18) note that the nutritional aspects they measured varied widely among microgreen types, providing fodder for future study. Additionally, Weber (22) noted that the methods used to grow microgreens (i.e., soil, compost, hydroponic) can significantly impact their nutritional value. A systematic comparison of the environmental impacts (i.e., water use, nutrient demand) of microgreen cultivation methods has not been conducted and should be considered alongside their impacts on nutritional value when deciding how to grow microgreens and if they are a nutrient-rich crop that can be sustainably produced.

In this study, the mineral concentration was determined for broccoli microgreens that had been grown hydroponically or using compost-based methods that are easily implemented by the average citizen. The nutritional value of the resulting microgreens was quantitatively compared to that of mature broccoli florets. In order to gain insights into the potential of local microgreen production to sustainably diversify food systems, particularly for urban areas, the nutritional value of microgreens was considered in the context of the resource demands (i.e., water, fertilizer, and energy inputs) of producing them relative to those of producing mature broccoli vegetable in California’s Central Valley.

## Materials and Methods

### Growing Microgreens

All growing and insert trays, humidity domes, and micro-mat Hydroponic Growing Pads used for growing microgreens were obtained from Handy Pantry (Salt Lake City, UT, USA). Five grams of broccoli seed (*Brassica oleracea* var. *Botrytis* Waltham 29; Mountain Valley Seeds, Salt Lake City, UT, USA) was sowed in each of 15, 5″ × 5″ insert trays containing vermicompost or micro-mat Hydroponic Growing Pads. The seeds in five insert trays containing vermicompost (C) and in five insert trays containing hydroponic growing pads (HW) were hydrated with sterile deionized water during the experiment; another set of five insert trays containing hydroponic growing pads (HFG) were hydrated with a 0.4% solution of General Hydroponics^®^ FloraGro^®^ Advanced Nutrient System^®^ 2-1-6 (GH Inc., Sebastopol, CA, USA), made in sterile deionized water. All 15-insert trays were placed into 10″ × 20″ black plastic growing trays for incubation; HFG and HW replicates were maintained in separate growing trays to avoid contaminating the HW replicates with the 0.4% FloraGro^®^ solution. After sowing, seeds were kept in the dark until germination (ca. 36 h) by covering the growing trays with aluminum foil. After germination, growing trays were covered with clear humidity domes and incubated under constant light produced by GE Plant and Aquarium Ecolux Bulbs. Bulbs were positioned ca. 6″ above the surface of the growth substrate creating a light intensity that ranged from 3,790 to 4,920 lux across the light field. During the growth period, insert trays were shifted randomly to different positions within the light field to ensure that the varied intensity across the light field did not adversely affect the experimental outcome. Sterile water or 0.4% FloraGro^®^ solution was applied as needed to the insert trays (10–25 mL volumes) during growth using sterile serological pipets to minimize the addition of microorganisms to the experiment. During the 7-day period from sowing to harvest, each HFG and HW replicate received a total of 90 mL of hydration and each C replicate received a total of 65 mL of hydration. Vermicompost was generated from coconut coir, kitchen scraps, and shredded paper using a Worm Factory and operating instructions from Uncle Jim’s Worm Farm (Spring Grove, PA, USA), 1 month prior to setting up growth experiments.

### Harvesting Microgreens

Microgreens were harvested 7 days after sowing using ethanol-cleaned scissors by cutting the cotyledon stems as close to the growth substrate as possible. Microgreens harvested from each of the 15 experimental replicates were placed into pre-weighed aluminum foil cups and then weighed immediately on a PB303-S/FACT Mettler Toledo analytical balance to determine the total harvested fresh weight in grams (gfw). From each experimental replicate, 0.091–0.110 gfw was placed into a protein extraction filter cartridge (see “protein analysis”) and 0.374–0.424 gfw was placed into 10 mL sterile conical tubes containing 5 mL of sterile 1× phosphate buffer (3.55 g L^−1^ Na_2_HPO_4_, 1.50 g L^−1^ KH_2_PO_4_) for washing microbes from the microgreen surfaces to determine microbial counts. The remaining biomass in the foil cups was placed into a drying oven at 80°C for 48 h, after which it was weighed again to determine the dry mass fraction.

### Elemental Analysis

Dried microgreens (2 g per each experimental replicate) were ground into a fine powder in a mortar and pestle and sent to the Penn State Agricultural Analytical Services Lab (University Park, PA, USA) for elemental analysis. There, using the methods of Huang and Schulte (23), each of the 15 samples was subjected to acid digestion procedures and analyzed using inductively coupled plasma optical emission spectrometry (ICP-OES) for quantitative measurement of the following elements: P, K, Ca, Mg, S, Na, Fe, Mn, Cu, Zn, Al, and B. For determination of total N concentration, each of the 15 samples was subjected to acid digestion and analysis on a Combustion Elementar Vario Max N/C Analyser using the methods of Horneck and Miller (24). For quality assurance/control in analyte measurements, the following were analyzed in conjunction with the samples using the same methods described above: standard reference material [e.g., NIST1515; expected value ±95% confidence interval (CI)], a laboratory quality control sample (one run for each analytical batch, expected value ±95% CI), continuing calibration verification standard (run after every 10 samples; expected value ±10%), method blank [must be less than the limit of quantification (LOQ)]. For total N measurements, the average mass of dried plant material subjected to analysis was 266 mg (range: 254–288 mg). For measurement of all other analytes using ICP-OES, the average sample mass subjected to analysis was 615 mg (range: 102–1,002 mg); the range of sample masses used was a function the limited amount of dried plant material that was available for each experimental replicate and the maximum amount possible was used for ICP-OES. For each analyte, the average method detection limit (MDL) and average LOQ, which are a function of the sample mass, were as follows: N [MDL: 0.034% (range: 0.031–0.035); LOQ: 0.188% (0.174–0.196)], P [MDL: 0.001% (0.0005–0.0031); LOQ: 0.003% (0.0012–0.0123)], K [MDL: 0.001% (0.0005–0.0049); LOQ: 0.007% (0.0025–0.0245)], Ca [MDL: 0.013% (0.005–0.049); LOQ: 0.033% (0.0124–0.0781)], Mg [MDL: 0.001% (0.0002–0.0016); LOQ: 0.003% (0.0010–0.0098)], S [MDL: 0.001% (0.0002–0.0025); LOQ: 0.007% (0.0025–0.0245)], Mn [MDL: 0.652 mg kg^−1^ (0.2478–2.4510); LOQ: 1.305 mg kg^−1^ (0.4955–4.9020)], Fe [MDL: 0.652 mg kg^−1^ (0.2480–2.4510); LOQ: 26.097 mg kg^−1^ (9.9108–98.0392)], Cu [MDL: 0.652 mg kg^−1^ (0.2478–2.4510); LOQ: 1.957 mg kg^−1^ (0.7440–7.3529)], B [MDL: 1.305 mg kg^−1^ (0.4955–4.9020); LOQ: 3.262 mg kg^−1^ (1.2401–12.2549)], Al [MDL: 2.610 mg kg^−1^ (0.9911–9.8039); LOQ: 6.524 mg kg^−1^ (2.4777–24.5098)], Zn [MDL: 0.652 mg kg^−1^ (0.2480–2.4510); LOQ: 6.524 mg kg^−1^ (2.4777–24.5098)], Na [MDL: 6.524 mg kg^−1^ (2.4777–24.5098); LOQ: 26.097 mg kg^−1^ (9.9108–98.0392)].

### Microbial Counts

Microgreens placed into conical tubes containing 5 mL of 1× phosphate buffer were incubated at room temperature on a LABQUAKE^®^ Rotisserie (Barnstead Thermolyne) for 45 min. Phosphate buffer containing microbes (100 μL) was serially diluted in 900 μL of 1× phosphate buffer, five times, for each of the 15 experimental replicates. From each of the 75-serial dilutions, 25 μL was spread-plated onto a tryptic soy agar (TSA; Sigma-Aldrich) plate and onto an Acumedia^®^ potato dextrose agar (PDA; DOT Scientific, Inc., Burton, MI, USA) plate for enumerating colony-forming units (CFUs). Plates were inverted and incubated for 48 h at room temperature. CFUs were counted on the lowest dilution plate containing a countable number of CFUs for each of the 15-experimental replicates.

### Data Analysis

Elemental analysis data and microbial counts for microgreens from the three growing treatments (HFG, HW, and C) were examined by the Shapiro Test for normality and the Fligner–Kileen Test for homoscedasticity using *R* software [version 3.2.2, R (25)]. Based on the results of these tests, a non-parametric Welch’s ANOVA (α = 0.05) followed by a Bonferroni Correction for multiple comparisons was utilized to determine if there were significant differences among the means for each of the three growing treatments with respect to microbial counts, protein concentrations, and elemental concentrations. The elemental concentration of microgreens was compared with that of mature, raw broccoli (vegetable) produced on industrial farms based on nutrient data in the USDA SR21 database (26).

## Results

### Biomass Yields

The harvested fresh mass in grams (gfw) differed significantly among the three growing treatments (*F*_2.000, 6.447_ = 17.8056, *P-*value = 0.002368). The average (*n* = 5) fresh mass of microgreens harvested from the HFG treatment (24.64 ± 0.32 gfw) was statistically greater than the average fresh mass harvested from the C treatment (20.00 ± 0.73 gfw, *P-*value = 0.0066) or the HW treatment (21.01 ± 1.23 gfw; *P-*value = 0.0310). The dry mass fraction for the three growing treatments ranged from 7.2 to 9.3%, falling within the same range noted for 25 different microgreens studied by Xiao et al. (18). The average dry masses (gdw) harvested from experimental replicates (*n* = 5) did not differ significantly among treatments (*F*_2.000, 5.671_ = 2.5156, *P*-value = 0.1652) and ranged from 1.53 to 1.96 gdw. The average water fraction (*n* = 5) for each of the growing treatments was as follows: C (0.913 ± 0.002), HFG (92.5 ± 0.1), and HW (91.0 ± 0.2).

### Element Concentration of Microgreens

**Figure 1:**
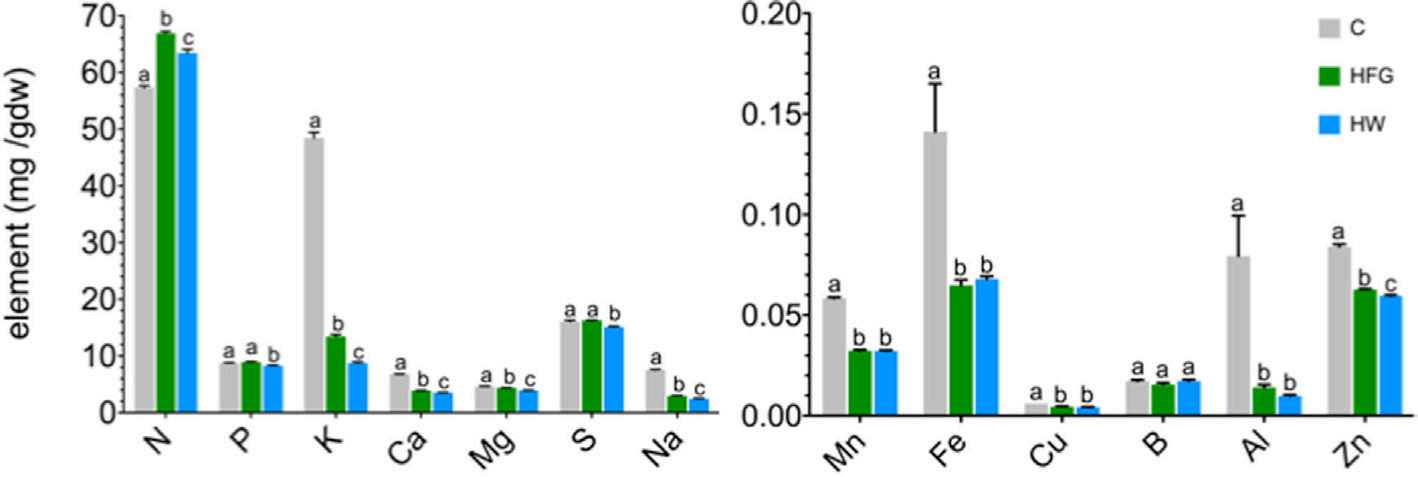
**Average (*n* = 5, ±1 SE) elemental concentration [mg (gdw)^−1^] of broccoli microgreens grown on compost (C), or hydroponically with a 0.4% solution of General Hydroponics^®^ FloraGro^®^ Advanced Nutrient System^®^ 2-1-6 (GH Inc., Sebastopol, CA, USA) (HFG) or with water only (HW)**. Note the differences in scale on the *y*-axes of the two graphs. Small letters denote statistically significant differences (α = 0.05); gdw, grams dry weight plant material.

The element concentrations of the microgreens are reported in Table 1 and are displayed in Figure 1. For all elements measured, except Fe and B, statistically significant differences were observed among the microgreens harvested from the three growing treatments (all *P-*values ≤0.01547). Differences in Fe concentration among the three growing treatments could be considered marginally significant (*F*_2.000, 6.368_ = 4.8853, *P*-value = 0.05177). Compost-grown microgreens had significantly greater amounts of K, Ca, Mg, Na, Zn, Mn, Fe, Cu, and Al than HFG or HW microgreens (Figure 1); Mn, Fe, Cu, Zn, and Al concentrations were statistically the same in HFG and HW microgreens (all *P-*values >0.08). HFG microgreens had significantly higher K (*P-*value = 4.7 × 10^−4^), Na (*P*-value = 1.8 × 10^−3^), N (*P*-value = 0.00063), P (*P*-value = 0.0004), Ca (*P*-value = 0.00084), Mg (*P*-value = 4.8 × 10^−6^), and S (*P*-value = 0.00082) than the HW microgreens (Figure 1). Nitrogen was the only element for which either the HFG or HW microgreens had a significantly higher concentration than the C microgreens (*P*-values ≤2.1 × 10^−6^; Figure 1).

**Table 1:** **Average (*n* = 5) element concentration [mg (gfw)^−1^] of broccoli microgreens grown on compost (C), or hydroponically with a 0.4% solution of General Hydroponics^®^ FloraGro^®^ Advanced Nutrient System^®^ 2-1-6 (GH Inc., Sebastopol, CA, USA) (HFG) or with water only (HW); gfw, grams fresh weight plant material**.

Element	Cultivation method					
	*C*		HFG		HW	
	mg (gfw)^−1^	SE	mg (gfw)^−1^	SE	mg (gfw)^−1^	SE
N	5.00	0.11	5.03	0.08	5.72	0.14
P	0.76	0.01	0.67	0.01	0.75	0.02
K	4.22	0.07	1.01	0.02	0.79	0.01
Ca	0.59	0.01	0.29	0.01	0.32	0.01
Mg	0.40	0.01	0.33	0.01	0.36	0.01
S	1.40	0.03	1.22	0.02	1.36	0.03
Na	0.66	0.01	0.22	0.0003	0.22	0.0005
Mn	5.09E−03	1.05E−04	2.42E−03	6.99E−05	2.90E−03	6.38E−05
Fe	1.25E−02	2.40E−03	4.87E−03	2.16E−04	6.12E−03	9.44E−05
Cu	5.23E−04	1.32E−05	3.31E−04	2.10E−05	3.79E−04	2.17E−05
B	1.50E−03	6.74E−05	1.16E−03	7.20E−05	1.55E−03	5.55E−05
Al	7.04E−03	1.94E−03	1.05E−03	9.96E−05	8.84E−04	5.74E−05
Zn	7.32E−03	1.28E−04	4.70E−03	7.77E−05	5.37E−03	1.20E−04

### Relative Nutritional Value of Broccoli Microgreens to Mature Vegetable

**Figure 2:**
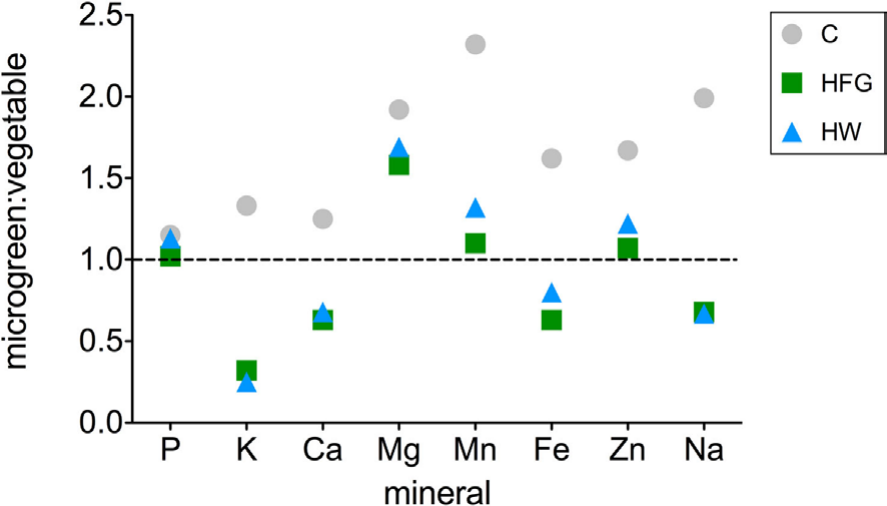
**Broccoli microgreen:vegetable mineral ratios for microgreens grown on compost (C) or hydroponically with a 0.4% solution of General Hydroponics^®^ FloraGro^®^ Advanced Nutrient System^®^ 2-1-6 (GH Inc., Sebastopol, CA, USA) (HFG) or with water only (HW)**. Data for raw broccoli florets (“vegetable”) were obtained from a published source (26). Ratios are reported only for the minerals which were reported for the mature vegetable; Cu was excluded because it was reported as 0 mg per serving for the mature vegetable (26). The horizontal line through one indicates equivalent mineral quantities in microgreens and vegetable.

Ratios of microgreen:broccoli vegetable (raw broccoli florets) mineral concentrations [mg element (gfw plant material)^−1^] are displayed in Figure 2 for P, K, Ca, Mg, Mn, Fe, Na, and Zn. The ratio for Cu is not reported because Cu was not detected in raw broccoli florets and was reported as 0 (26). Microgreen:broccoli vegetable ratios for C microgreens for all minerals examined ranged from 1.15 to 2.32. For HFG and HW microgreens, microgreen:broccoli vegetable ratios were ≥1.01 for all minerals except for K, Ca, Na, and Fe, which ranged from 0.25 to 0.80. The average microgreen: broccoli vegetable ratios for C, HFG, and HW treatments were 1.73, 0.86, and 0.95, respectively.

### Microbial Counts

**Figure 3:**
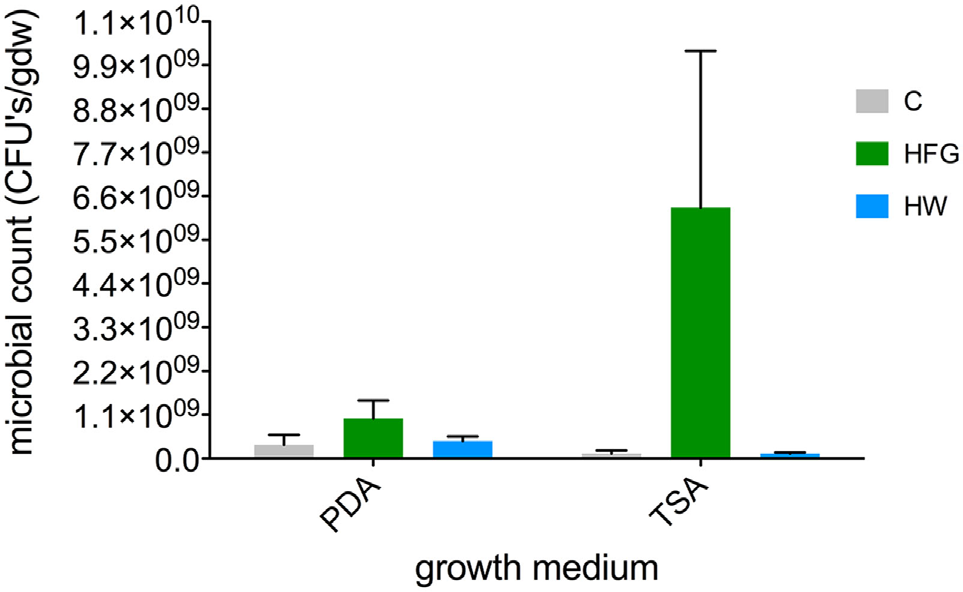
**Average (*n* = 5, ±1 SE) microbial counts [CFUs (gdw)^−1^] on two different kinds of microbial growth media (TSA, PDA) for broccoli microgreens grown on compost (C) or hydroponically with a 0.4% solution of General Hydroponics^®^ FloraGro^®^ Advanced Nutrient System^®^ 2-1-6 (GH Inc., Sebastopol, CA, USA) (HFG) or with water only (HW)**. CFUs, colony-forming units, gdw, grams dry weight plant material, TSA, tryptic soy agar, PDA, potato dextrose agar.

Of the three growth treatments, microbial counts (per gdw plant material) for HFG microgreens were the greatest with average counts of 1.24 × 10^8^ ± 7.83 × 10^7^ on TSA and 3.46 × 10^8^ ± 2.51 × 10^8^ on PDA. However, due to large variability among replicates, these counts were not statistically greater than average counts observed on either medium type for C microgreens (TSA = 1.24 × 10^8^ ± 7.83 × 10^7^; PDA = 3.46 × 10^8^ ± 2.51 × 10^8^) or HW microgreens (TSA = 1.24 × 10^8^ ± 2.73 × 10^7^; PDA = 4.37 × 10^8^ ± 1.15 × 10^8^) (Figure 3; all *P-*values ≥0.3882).

## Discussion

Although the three different cultivation methods used in this study substantially impacted the nutritional value of the resulting microgreens, results demonstrated that broccoli microgreens may have superior nutritional value to the mature vegetable with respect to several of the minerals examined. Regardless of how they were grown, microgreens had larger quantities of Mg, Mn, Cu, and Zn than the vegetable (Figure 2). However, compost-grown (C) microgreens had higher P, K, Mg, Mn, Zn, Fe, Ca, Na, and Cu concentrations than the vegetable. For eight minerals analyzed that are commonly reported in nutrition information facts for foods (P, K, Ca, Mg, Mn, Fe, Zn, and Na), the average C microgreen: vegetable nutrient ratio was 1.73. In contrast, the average ratios for these minerals for hydroponically grown microgreens were only 0.86 (HFG) and 0.95 (HW), indicating that they had slightly less nutritional value than the vegetable (Figure 2). Assuming that C microgreens are 1.73 times more nutritious than the vegetable on a per gfw basis, one would need to eat ca. 42% less mass of microgreens (ca. 53 gfw) to obtain the same amount of minerals present in a serving of raw broccoli florets [91 g (26)].

The relatively high nutritional value of broccoli microgreens compared to the vegetable is consistent with previous studies reporting that produce at early growth stages (i.e., sprouts, microgreens, “baby” vegetables) are denser sources of nutrition than their mature counterparts (27–29). It has been noted that vegetables, especially when grown on nutrient poor soils, have low mineral concentrations. Fertilization of nutrient poor soils can increase mineral concentration in plant leaves, but not always in the produce that is consumed because minerals are not distributed evenly in all plant parts (30). In the case of cereals, milling or polishing tend to further deplete the nutritional value of cereals and grains, removing the relatively Zn, Fe, and Cu-rich bran (30). For example, the iron concentration of a rice leaf is generally 100–200 ppm, but is only ca. 3 ppm in the polished rice grain that is consumed (31). Therefore, simply increasing fertilizer application does not represent a viable solution for improving the nutritional value of crops and simultaneously has negative consequences on the environment. Additionally, fertilizer manufacturing is no longer sustainable at current rates (32). In this context, the potential to grow microgreens themselves without the use of fertilizer application is intriguing. However, the potential of microgreen production to reduce the overall fertilizer application rates for commercial broccoli vegetable production will depend on (1) how much broccoli vegetable production for the purposes of consumption can be replaced by microgreen production and (2) how the scale of broccoli production required for seed production for microgreen cultivation will change. These factors will depend on how the consumer demand for microgreens vs. the mature vegetable will change, which is unknown at this point in time.

The cultivation methods utilized in this study significantly impacted the elemental concentration of the microgreens. With respect to the 13 elements analyzed, C microgreens had significantly greater quantities of nine elements (K, Ca, Mg, Na, Mn, Fe, Cu, Al, and Zn) than the hydroponically grown microgreens (all *P-*values ≤0.0086). Relative to the HW microgreens, HFG microgreens had significantly greater quantities of only seven of the elements (N, P, K, Ca, Mg, S, and Na; all *P-*values ≤0.0018). These results likely reflect the differing availabilities of the nutrients in the growth substrate, which were not equalized across growing methods in this study. In choosing the three growing methods tested in this study, the goal was to utilize cultivation methods that can be applied easily for growing microgreens in one’s home and have been recommended by popular sources. For instance, growing microgreens hydroponically using a 0.4% solution of FloraGro is recommended in one vendor’s educational resources (https://www.growingmicrogreens.com). While HFG microgreens had larger quantities of N, P, and K than HW microgreens, which was expected with the application of a fertilizer containing those elements, HFG and HW microgreens had similar quantities of other key nutritional elements (Mn, Fe, Cu, and Zn). Treadwell et al. (19) note that growing many microgreen varieties themselves (not considering growth of mature vegetable plants for the purposes of seed production) may not require any fertilizer because the seed provides enough nutrition to fuel growth to the cotyledon stage and, therefore, adding fertilizer will not increase mineral concentration.

In addition to C microgreens having superior nutrition to HFG and HW microgreens, utilizing compost as a growth substrate can help close nutrient loops by reducing waste that ends up in landfills, where it produces large amounts of greenhouse gases (GHGs). The compost used to grow C microgreens in this study was generated using a small vermicomposter that can be easily managed inside someone’s home, even if it is a small urban dwelling. Composted materials included “unavoidable waste” from fruit and vegetables that are nutrient rich, but go uneaten (i.e., avocado and banana peels). Growing microgreens in the resulting vermicompost provided a mechanism for recapturing some of these nutrients in plant biomass for human consumption rather than having it lost to a landfill. For example, a banana peel composes ca. 40% of the fresh weight of the whole fruit (33), but it gets thrown away as municipal waste despite being rich in dietary fiber, proteins, amino acids, polyunsaturated fatty acids, and potassium (34). Poor soil quality is a primary driver of malnourishment in plant-based diets (15, 16) and has triggered the breeding of genetically modified crops that can efficiently sequester nutrients from such soils (30), as well as the increased dependence on fertilizer application, which is no longer sustainable at current rates (32). However, as demonstrated in this study, nutrient-rich microgreens can be grown on compost in the absence of genetic engineering and fertilizer application. Although one recognized advantage of hydroponic growing methods is their lack of dependence on soil (35), it should be noted that composting methods also depend on very little soil starter and, as data here show, can be used to grow microgreens that are much more nutrient-rich than hydroponic methods.

Relative to field-based cropping methods, greenhouse-based hydroponic growing methods are advantageous in that they require little machinery and pesticide use, make farming possible in non-arable lands, and produce vegetables of higher quality and yield (35). However, greenhouse operation contributes to about 74% of agriculture’s total energy use and contributes about one-third of the total GHG emissions (35). In contrast, if microgreens are cultivated in private homes in a distributed agricultural model, they likely would not require much more energy to cultivate than people usually use to power their homes, especially if natural sunlight was used rather than grow lights. Indoor growth as well as fast generation times, also protects microgreens from pests, minimizing the need to apply environmentally harmful pesticides (2). That said, it should be noted that microbial counts were higher, on average, for hydroponically grown microgreens than on C microgreens, indicating that hydroponically grown microgreens may be more susceptible to microbial contamination. Collectively, these insights bolster the case for growing microgreens on compost using a distributed agricultural model rather than industrial greenhouse-based hydroponic growing methods.

Water consumption is another critical aspect to consider in assessing the sustainability of cropping methods, as water is being utilized in some regions faster than the natural recharge of their aquifers [e.g., midwestern U.S., Ganges Plains, and Northern China Plains (2)]. About 70% of global water use is related to agriculture and, annually, 2,600 km^3^ of water are used to irrigate crops across the globe, representing 2/3 of human water withdrawals (36). Water use is only likely to increase as the population grows along with the demand per capita of water use as household incomes increase and diets shift toward more water-demanding products (37). Extrapolation from experimental data presented here indicates that broccoli microgreens would require 158–236 times less water than it does to grow a nutritionally equivalent amount of broccoli vegetable in fields in California’s Central Valley. The broccoli yield per acre in California’s Central Valley is ca. 18,400 lbs [800 boxes weighing 23 lbs each (38)], which equates to ca. 91,715 servings [91 g per serving (26)]. On the basis that C microgreens are 1.73 times more nutritious than the mature vegetable, production of 10,635 lbs of microgreens would provide the same amount of nutrition as a one-acre broccoli field in the Central Valley. Based on the C microgreen yields and water application rates in this study, only 15,679 L of water would be needed compared to the 2,480,000–3,700,000 L applied per acre of broccoli in the Central Valley (38).

Broccoli production on industrial farms takes 100–150 days in California’s Central Valley (38), but growing microgreens indoors takes 7–9 days, depending on growing conditions, from sowing to harvest. This represents a 93–95% reduction in production time, which is especially intriguing given the needs to ramp up food production efficiency to feed a growing population. In order to sustain broccoli microgreen production, broccoli plants still need to be grown to maturity out in the fields for the purpose of seed production. The amount of seed that would be required to produce broccoli microgreens as a primary food crop remains a question. This would partially depend on the scale at which broccoli microgreens were going to be produced in conjunction with defining an optimal density at which they should be planted. It is generally recommended that seeds are planted at high density, blanketing the growth substrate, in order to grow them as microgreens, but more work is necessary to determine an optimal density for planting seeds in order to maximize nutrient uptake (i.e., nutritional value of produce) and harvest yield.

In addition to activities associated with crop production, another factor that significantly impacts the nutritional value of produce in markets and contributes to environmental damage, particularly in the form of GHG emissions, is the length of time it takes for produce to reach food markets post-harvest. In the 1900s, most food in the U.S. was produced and consumed locally (39–41) but now transported thousands of miles from farm to market. For instance, produce sold at a food market in Chicago, IL, USA travels an average of 1,500 miles prior to arrival (40). Such transport is not only an energy-demanding and GHG-producing process but also increases the duration between harvest and consumption of produce, which can diminish its nutritional value. For instance, it has been documented that fresh peas stored at ambient temperatures lost 50% of their ascorbic acid in 7 days and spinach can lose 100% of its ascorbic acid in less than 4 days (12). Losses can be reduced for some crops by storing and transporting them at cooler temperatures, but refrigeration can negatively impact the environment. For instance, Carlsson-Kanyama (42) noted that 60% of the total greenhouse gas emissions resulting from carrot production could be attributed to their long-term cold storage in facilities where leakage of refrigerants is known to occur. Microgreens have a very short-shelf life and their production at industrial scales and subsequent distribution would also require cold-transport and storage. As seedlings predominately respire during germination and carbohydrates are rapidly depleted in their cells, they wither rather quickly (43). At 4°C, the shelf life for some microgreens might be 14–21 days, but a mere 6° increase can reduce shelf life by 50% (43). Studies are underway to identify ways to extend the shelf life of microgreens [i.e., addition of CaCl_2_ (44)], which could make transport more feasible. This is certainly exciting for commercial outfits looking to cultivate microgreens, which may be able to vie for a significant fraction of the $500 million sprouts market (45). However, growing microgreens *via* distributed agricultural methods, in which they are grown and consumed locally, eliminates the need for long-distance transport, reducing fossil fuel consumption, and provides consumer-access to more nutrient dense produce. Local production and consumption of microgreens may also prevent them from contributing to the 30–40% of produce that is currently being lost annually during transport from farm to market and, therefore, GHG-production when this waste is sent to landfills (2). In the U.S., uneaten food is the largest component of municipal solid waste and is responsible for a large portion of the country’s total methane emissions (3).

Additionally, in contrast to mature vegetables, microgreens generate little to no food waste during meal preparation and cooking. For example, in the UK, it is reported that a total of 4.1 million tonnes of food is thrown away on an annual basis that is avoidable with better management and food preparation strategies; 1.3 million tonnes of total food waste (19% of the total food waste) is “unavoidable waste” (e.g., vegetable peelings, meat carcasses, teabags). In the case of broccoli vegetable, many people eat only the florets and discard the stems even though the stems are perfectly edible and have large quantities of antioxidants (46). However, none of the microgreen biomass is wasted through trimming, as whole cotyledons are harvested at the surface of the growth substrate for consumption; therefore, with the exception of the roots, 100% of the microgreen biomass generated can be consumed.

Microgreen cultivation by individuals is a potential mechanism to diversify food systems, which is necessary to increase society’s resilience to environmental change. However, diversifying food systems by any mechanism requires generating public awareness of the pitfalls of current food systems and, ultimately, people altering their behavior and assuming political and individual responsibility (2). The concentration of people in metropolitan areas is certainly a driving force behind the long distance transport of food from farms to metropolitan centers. Such long-distance transport is also driven by the prevailing attitude of an increasingly global society that expects a diversity of food products to be available year-round (2). Cultivation of microgreens in individual households could make some progress in combating food deserts un urban areas but requires educating the masses on food system problems, how that impacts nutrition, and how their altered behavior (i.e., becoming more self-sufficient) can contribute to the solution.

Empowering more individuals with mechanisms to increase the resilience of food systems in urban areas poses several advantages over current urban agriculture efforts. The creation of multifunctional land spaces in urban areas for cultivating food have made some progress in improving food system sustainability, increasing nutritional resources in food deserts, decreasing food packaging and processing, decreasing GHG emissions compared with conventional food systems, and improving waste management (e.g., composting, waste water recycling). However, there are a number of challenges associated with creating community gardens in urban spaces. These challenges include: access to land that is suitable for food production, proximity of space to running water, protection of space from vandalism and theft, accessibility for gardeners, proximity to market, lack of sufficient supportive services and infrastructure, and discrepancies between the actual and perceived health risks of growing food in an urban environment (47). Additionally, there are concerns in diverse communities that such spaces serve individuals rather than the public at large, as different socioeconomic and demographic groups place different values on various land uses and functionalities (47). Growing microgreens at home allows individuals to take complete responsibility of the growing process and conditions. This eliminates some of the concerns that are difficult to satisfy in community efforts, the need for support staff and infrastructure, and places production and consumption in the same location. The ease with which even inexperienced gardeners can grow microgreens in urban dwellings has the potential to empower individuals to take responsibility of generating some of their own food, given that they are made aware of the benefits of doing so and undergo attitudinal and behavioral changes to realize this solution.

## Conclusion

This study provides critical insights into the potential for broccoli microgreens to provide a dense source of minerals that can be grown with a small ecological footprint by individuals in a distributed agricultural model. Microgreen production could also diversify the average diet, as broccoli is only one of many nutrient-rich microgreens that can be easily produced and consumed by individuals (22). Therefore, with proper education of the general public and subsequent action, microgreen production and consumption represents a viable mechanism for diversifying food production systems, which is necessary for increasing societal resilience to environmental changes that threaten long industrial food chains. Although community gardens have made some headway and successful ones should not be abandoned, microgreens have the advantage of empowering individuals to take responsibility without the need for extensive community networking and infrastructure development.

## Author Contributions

The author designed and executed the research detailed within and wrote the manuscript.

## Conflict of Interest Statement

The author declares that the research was conducted in the absence of any commercial or financial relationship that could be construed as a potential conflict of interest.
